# Characteristics of older adults receiving enteral feeding at a geriatric medical center

**DOI:** 10.1186/s12877-024-05202-y

**Published:** 2024-07-23

**Authors:** Galina Plotnikov, Yochai Levy, Daniel Trotzky, Ahmad Nassar, Yosef Bushkar, Estela Derazne, Dana Kagansky, Miya Sharfman, Nadya Kagansky

**Affiliations:** 1 Faculty of Medicine, Tel Aviv University, Tel Aviv, Israel; 2 Shmuel Harofe Geriatric Medical Center, Be’er Ya’akov, Israel; 3 Yitzhak Shamir Medical Center, Zerifin, Israel

**Keywords:** Enteral feeding, Enteral nutrition (EN), Geriatric, Long- term, Mortality, Nasogastric tube feeding, Older adults

## Abstract

**Background:**

Malnutrition is a prevalent and hard-to-treat condition in older adults. enteral feeding is common in acute and long-term care. Data regarding the prognosis of patients receiving enteral feeding in geriatric medical settings is lacking. Such data is important for decision-making and preliminary instructions for patients, caregivers, and physicians. This study aimed to evaluate the prognosis and risk factors for mortality among older adults admitted to a geriatric medical center receiving or starting enteral nutrition (EN).

**Methods:**

A cohort retrospective study, conducted from 2019 to 2021. Patients admitted to our geriatric medical center who received EN were included. Data was collected from electronic medical records including demographic, clinical, and blood tests, duration of enteral feeding, Norton scale, and Short Nutritional Assessment Questionnaire score. Mortality was assessed during and after hospitalization. Data were compared between survivors and non-survivors. Multivariate logistic regressions were performed to identify the variables most significantly associated with in-hospital mortality.

**Results:**

Of 9169 patients admitted, 124 (1.35%) received enteral feeding tubes. More than half of the patients (50.8%) had polypharmacy (over 8 medications), 62% suffered from more than 10 chronic illnesses and the majority of patients (122/124) had a Norton scale under 14. Most of the patients had a nasogastric tube (NGT) (95/124) and 29 had percutaneous endoscopic gastrostomies (PEGs). Ninety patients (72%) died during the trial period with a median follow-up of 12.7 months (0.1–62.9 months) and one-year mortality was 16% (20/124). Associations to mortality were found for marital status, oxygen use, and Red Cell Distribution Width (RDW). Age and poly-morbidity were not associated with mortality.

**Conclusion:**

In patients receiving EN at a geriatric medical center mortality was lower than in a general hospital. The prognosis remained grim with high mortality rates and low quality of life. This data should aid decision-making and promote preliminary instructions.

## Introduction

The healthcare landscape has undergone remarkable progress, resulting in a notable increase in the global population of older adults. Projections suggest that by 2050, people aged 60 or above will make up nearly 22% of the total population, compared to approximately 12% in 2015 [1]. As individuals age, a range of physiological, functional, and cognitive processes along with medical aspects leads to various nutritional considerations. This includes decreased metabolic rate and potential support requirements for feeding-related actions [2]. Nutritional inadequacy can result from factors such as alterations in sensory perception, shifts in energy needs, reduced physical activity, muscle loss, environmental and financial constraints, and psychosocial factors [3]. These changes contribute to malnutrition becoming a prevalent issue among older adults, with rates ranging from 12 to 50% in hospitalized cases and from 23 to 60% among institutionalized older adults [4, 5].

A diet alone might not be enough to meet the age-specific requirements of older adults, and enteral feeding might be necessary to bolster nutritional status in several specific conditions such as dysphagia, dementia, and else [6–8]. Although there is limited evidence of its benefits, in the absence of alternatives tube feeding remains prevalent in the treatment of malnourished older adults [9].

Analyzing the mortality rate and the influential factors among older adult patients admitted to geriatric departments with enteral feeding or starting enteral feeding can significantly aid physicians, patients, and families in making informed decisions about their care and management. A prior study, showed that among older acutely ill patients in a general hospital initiated on NGT feeding, high in-hospital mortality was observed. Pressure sores, lymphopenia, and lower serum cholesterol levels were the most significant contributing factors [10].

This paper aims to continue assessing variables and in-hospital mortality among older patients with enteral feeding admitted to the geriatric hospital.

## Materials and methods

### Study population and design

A cohort retrospective study was conducted at the Shmuel Harofe Geriatric Medical Center, in Israel, from January 2019 to December 2021. The center has 9 departments, including internal medicine, rehabilitation, skilled nursing care for severely ill patients, and chronic mechanical ventilation departments. All patients admitted to the geriatric center underwent a comprehensive geriatric assessment including a geriatrician, nurse, social worker, and dietician. A speech therapist evaluated all patients in need of new enteral feeding. Additional evaluation by a physiotherapist or occupational therapist was provided when needed. Inclusion criteria included admission to the geriatric center during the mentioned period and enteral feeding that began before or during hospitalization.

### Enteral feeding

Enteral feeding was given by Nasogastric Tube (NGT) or via percutaneous endoscopic gastrostomy (PEG).

The hospital did not hospitalize acute surgical or ICU patients; therefore, most patients received enteral feeding due to chronic medical conditions. These were mainly neurologic deficits due to advanced dementia, prior stroke, or other chronic conditions resulting in swallowing problems with either low intake, malnutrition, dehydration, or recurrent aspirations. In our hospital, all patients were assessed by a dietician, an expert in geriatric medicine physician, and if necessary, a speech therapist. This multidisciplinary approach allowed for re-assessing the need for enteral, adjusting caloric and protein intake, and managing complications. All enteral feeding was administered by highly trained nursing staff under supervision.

### Data collection

Data was collected from the electronic medical center records (EMRs), including demographic, clinical, and blood test results, blood cell count, albumin, and electrolytes. Relevant nutritional data including the cause of hospitalization, chronic illness, the date and cause of tube insertion, type of inserted tube, NGT or percutaneous endoscopic gastrostomy (PEG), type of feeding tube formula, duration of EN, Norton scale and Short Nutritional Assessment Questionnaire (SNAQ) score were also collected. A participant flow chart was provided to show the flow of the study.

### Norton scale

General status was assessed by the Norton Scale. The patients were divided according to the risk categories of the Norton scale [11]. The Norton Scale is a well-validated tool for assessing the risk of pressure sore development [12]; however, in recent years this scale is frequently been used for the assessment of general cognition because the scale includes cognitive and functional assessment [13]. Additionally, pressure sore development reflects the general, functional, and cognitive decline in the patient’s status [14]. The Norton Scale, a pressure ulcer risk assessment tool, encompasses five elements: physical condition, mental health, activity, mobility, and incontinence. The five subscale scores of the Norton Scale are added together for a total score that falls between 5 and 20. Patients are categorized based on their Norton Scale scores into four groups: Norton < 10 (very high risk), 10–14 (high risk), 14–18 (medium risk), and Norton > 18 (low risk).

### Short nutritional assessment questionnaire (SNAQ)

SNAQ score [15, 16] is a validated tool used for malnutrition risk assessment encompassing questions about weight loss, appetite, and food intake. Patients were categorized based on their SNAQ score (between 0 and 5) into two main groups, SNAQ < 0–2 indicating low risk for malnutrition, and SNAQ > 2 with suspected malnutrition. Patients with SNAQ scores of > 2 completed their nutrition assessment with the intervention of a dietician. The primary outcome was overall mortality, with the follow-up period extending from the beginning of enteral feeding until the end of the trial period on April 22, 2022. Secondary outcomes included one-year mortality and mortality during hospitalization. Mortality data was collected using both the hospital’s electronic medical records (EMRs) for in-hospital mortality and a national database available to hospitals for out-of-hospital mortality.

### Statistical analysis

The distribution of sociodemographic characteristics at admission was calculated for all study populations. Because blood test results were not normally distributed, median, 25th, and 75th percentiles were presented. The association between risk factors and mortality was assessed with Cox proportional hazard models, hazard ratios (HR), 95% confidence intervals (95%CI), and p-values were presented. Log minus log figures were inspected and confirmed the proportionality of the hazard. Collinearity among the variables introduced in multivariable analysis (sex, age at feeding initiation, marital status, pressure ulcer, hypothyroidism, oxygen use, fever, cause of hospitalization: sepsis and neoplasm) was examined, with the maximum Variance Inflation Factor (VIF) = 1.525. A backward stepwise method with probability in = 0.05 and probability out = 0.10 was used for variable selection in multivariable analysis. Statistical analyses were performed with IBM SPSS Statistics for Windows, version 29.0.1.0 Armonk, NY: IBM Corp. A two-sided p-value ≤ 0.05 was considered statistically significant.

## Results

**Fig. 1:**
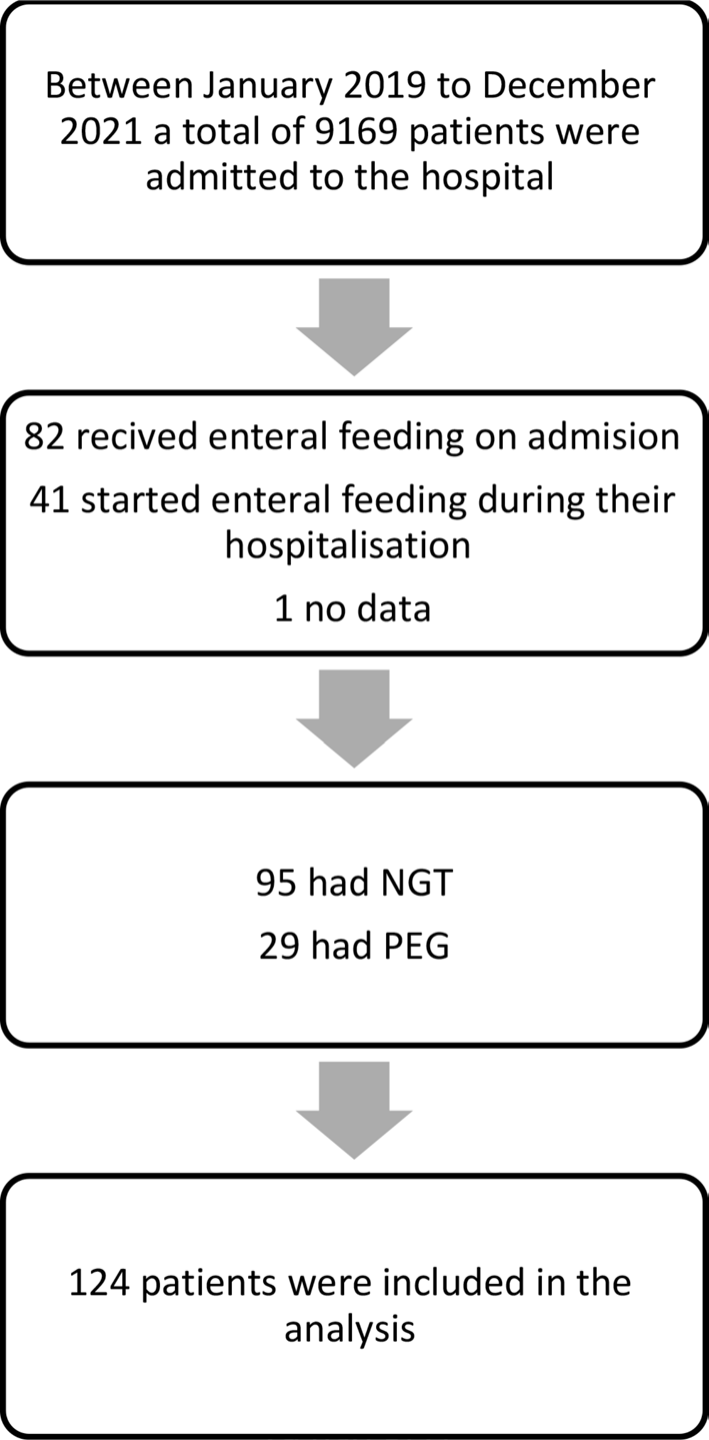
Participant flow chart. *Abbreviations* NGT, Nasogastric tube; PEG, Percutaneous endoscopic tube

Between January 2019 to December 2021, a total of 9169 patients were admitted to the hospital. One hundred and twenty-four (1.35%) of them received enteral feeding tubes which were inserted during or before hospitalization. Figure 1 presents an overview of the participant flow throughout the study. During which, 66.9% of the patients admitted to our medical center arrived with a pre-existing feeding tube and 33.1% had one inserted during their hospitalization. The follow-up duration ranged from a minimum of 0.1 months to a maximum of 62.9 months, with a median duration of 12.7 months and a total cumulative duration of 1911 patient-months. Most patients had a NGT (95/124) and 29 had PEGs. Their ages ranged from 56 to 104 with a median age of 85 years, and 71 (57.3%) were females.

Of the 124 patients receiving enteral feeding, during the study period 90 (76%) patients died, and 49 of them died during hospitalization. Most patients (82/124) started enteral feeding before their hospitalization. Of these 82 patients, 10 died during the first year and 53 died during the study period.

**Fig. 2:**
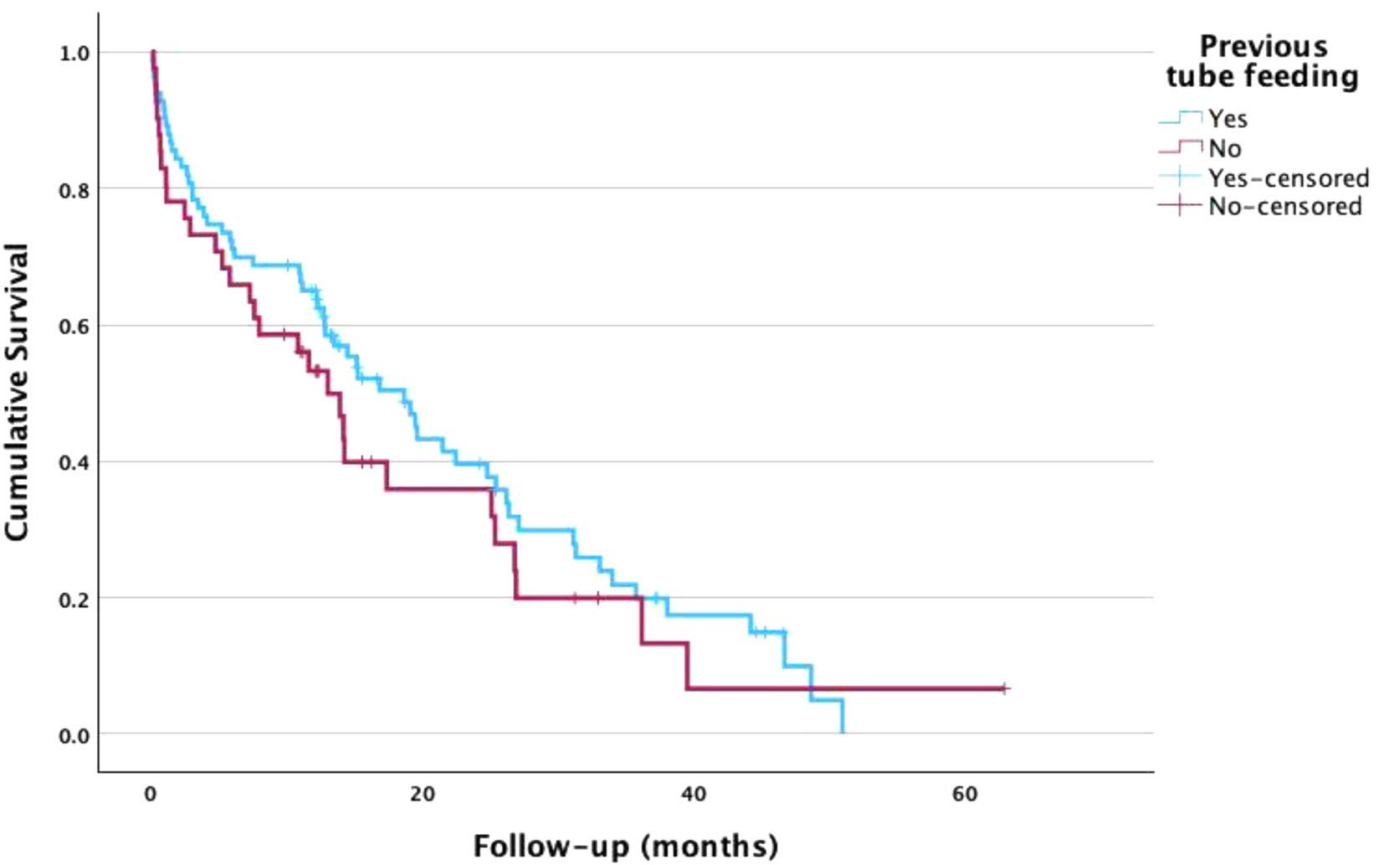
Kaplan-Meier cumulative survival curves for patients receiving tube feeding before or during hospitalization

Table 1 presents demographic and clinical characteristics and their association with mortality rate in a univariate analysis. A significant correlation was found between marital status and mortality rates. Specifically, divorced and widowed individuals exhibited a higher mortality rate (HR = 2.60, 95% CI [1.19–5.70], *p* = 0.017 and HR = 1.56, 95% CI [0.98–2.49], *p* = 0.062 respectively) compared to their married counterparts. Conversely, factors such as sex and number of children did not yield statistically significant differences in the mortality rate. Furthermore, examining the causes of hospitalization, patients admitted due to neoplasm disease (*p* = 0.018) and sepsis (*p* = 0.011) exhibited significantly higher mortality rates compared to those with other causes, however, the number of patients with these causes for hospitalization was very low. Factors such as CVA, fever, pneumonia, UTI, cellulitis, and pressure ulcers did not yield statistically significant differences in the mortality rate. In terms of Norton scores, none of the categories showed statistically significant differences in mortality rates, however, most patients (122 of 124) had low Norton scale scores (lower than 14). Most patients (97/124) had a SNAQ score of 2 or less. Neither the SNAQ score nor weight loss showed a statistically significant effect on the mortality rate (p-value 0.83 for SNAQ score and 0.6 for weight loss), nor did the type of inserted tube (PEG or NGT) or type of nutrition formula. Additionally, the number of chronic illnesses and the number of medications (polypharmacy) did not have a significant effect. Among the chronic illnesses assessed, pressure ulcers (HR = 1.76, 95% CI [1.08–2.88], *p* = 0.024), oxygen use (HR = 1.60, 95% CI [1.05–2.45], *p* = 0.030), and fever (HR = 1.50, 95% CI [0.99–2.26], *p* = 0.056) showed significant associations with mortality rates. Conversely, hypothyroidism, chronic obstructive pulmonary disease (COPD), diabetes mellitus (DM), delirium, and consciousness did not exhibit statistically significant differences in the mortality rates. In terms of TSH levels, none of the categories showed statistically significant differences in the mortality rate. No differences were found between patients receiving EN prior to their hospitalization and those with new EN (Table 1; Fig. 2).

**Table 1:** Association of demographic and clinical characteristics with mortality: univariate survival analysis results

	Total		Alive		Death			95% CI		
	*N*	(%)	*N*	(%)	*N*	(%)	HR	lower	upper	*P*
Total	124	(100.0)	34	(100.0)	90	(100.0)				
Men	53	(42.7)	13	(38.2)	40	(44.4)	1.29	0.85	1.96	0.227
Women	71	(57.3)	21	(61.8)	50	(55.6)	1.00			
Marital Status										0.041
Single	6	(4.8)	1	(2.9)	5	(5.6)	0.75	0.29	1.96	0.560
Married	47	(37.9)	15	(44.1)	32	(35.6)	1.00			
Widowed	63	(50.8)	18	(52.9)	45	(50.0)	1.56	0.98	2.49	0.062
Divorced	8	(6.5)	0	(0.0)	8	(8.9)	2.60	1.19	5.70	0.017
Children										0.200
no	7	(5.6)	1	(2.9)	6	(6.7)	1.00			
1–3	102	(82.3)	30	(88.2)	72	(80.0)	1.73	0.74	4.03	0.207
> 3	15	(12.1)	3	(8.8)	12	(13.3)	1.10	0.41	2.96	0.845
Tube feeding										
Before Hosp.	83	(66.9)	23	(67.6)	60	(66.7)	1.00			
During Hosp.	41	(33.1)	11	(32.4)	30	(33.3)	1.23	0.79	1.91	0.357
Formula										0.471
Easy Fiber	79	(63.7)	26	(76.5)	53	(58.9)	1.00			
Easy Shake	4	(3.2)	1	(2.9)	3	(3.3)	1.22	0.38	3.94	0.735
Easy Mealk	18	(14.5)	2	(5.9)	16	(17.8)	1.40	0.79	2.46	0.250
Other	23	(18.5)	5	(14.7)	18	(20.0)	1.46	0.85	2.51	0.175
C/H CVA	7	(5.6)	4	(11.8)	3	(3.3)	0.88	0.28	2.81	0.829
C/H Fever	8	(6.5)	2	(5.9)	6	(6.7)	1.51	0.66	3.46	0.335
C/H Pneumonia	30	(24.2)	9	(26.5)	21	(23.3)	1.28	0.78	2.11	0.330
C/H UTI	11	(8.9)	4	(11.8)	7	(7.8)	1.11	0.51	2.41	0.795
C/H Cellulitis	2	(1.6)	0	(0.0)	2	(2.2)	0.87	0.21	3.60	0.853
C/H Sepsis	2	(1.6)	0	(0.0)	2	(2.2)	6.62	1.53	28.58	0.011
C/H Feeding Problems	11	(8.9)	5	(14.7)	6	(6.7)	1.64	0.70	3.81	0.255
C/H Neoplasm	7	(5.6)	1	(2.9)	6	(6.7)	2.77	1.19	6.43	0.018
C/H Pressure Ulcer	41	(33.1)	4	(11.8)	37	(41.1)	1.07	0.70	1.64	0.762
C/H Others	34	(27.4)	13	(38.2)	21	(23.3)	0.74	0.45	1.21	0.233
chronic illnesses > 10	77	(62.1)	16	(47.1)	61	(67.8)	1.21	0.77	1.88	0.408
Medication > 8	63	(50.8)	19	(55.9)	44	(48.9)	0.96	0.64	1.46	0.867
COPD	21	(16.9)	3	(8.8)	18	(20.0)	0.71	0.42	1.20	0.203
Pressure Ulcers	83	(66.9)	14	(41.2)	69	(76.7)	1.76	1.08	2.88	0.024
Hypothyroidism	30	(24.2)	14	(41.2)	16	(17.8)	0.60	0.35	1.03	0.063
S/Oxygen use	41	(33.1)	5	(14.7)	36	(40.0)	1.60	1.05	2.45	0.030
S/DM	70	(56.5)	22	(64.7)	48	(53.3)	1.29	0.85	1.97	0.234
S/Delirium	28	(22.6)	3	(8.8)	25	(27.8)	1.26	0.79	2.00	0.333
S/Fever	50	(40.3)	4	(11.8)	46	(51.1)	1.50	0.99	2.26	0.056

*Abbreviations* C/H, cause of hospitalization; COPD, chronic obstructive pulmonary disease; CVA, cerebro-vascular accident; DM, diabetes mellitus; S/, symptoms during hospitalization; UTI, urinary tract infection

**Fig. 3:**
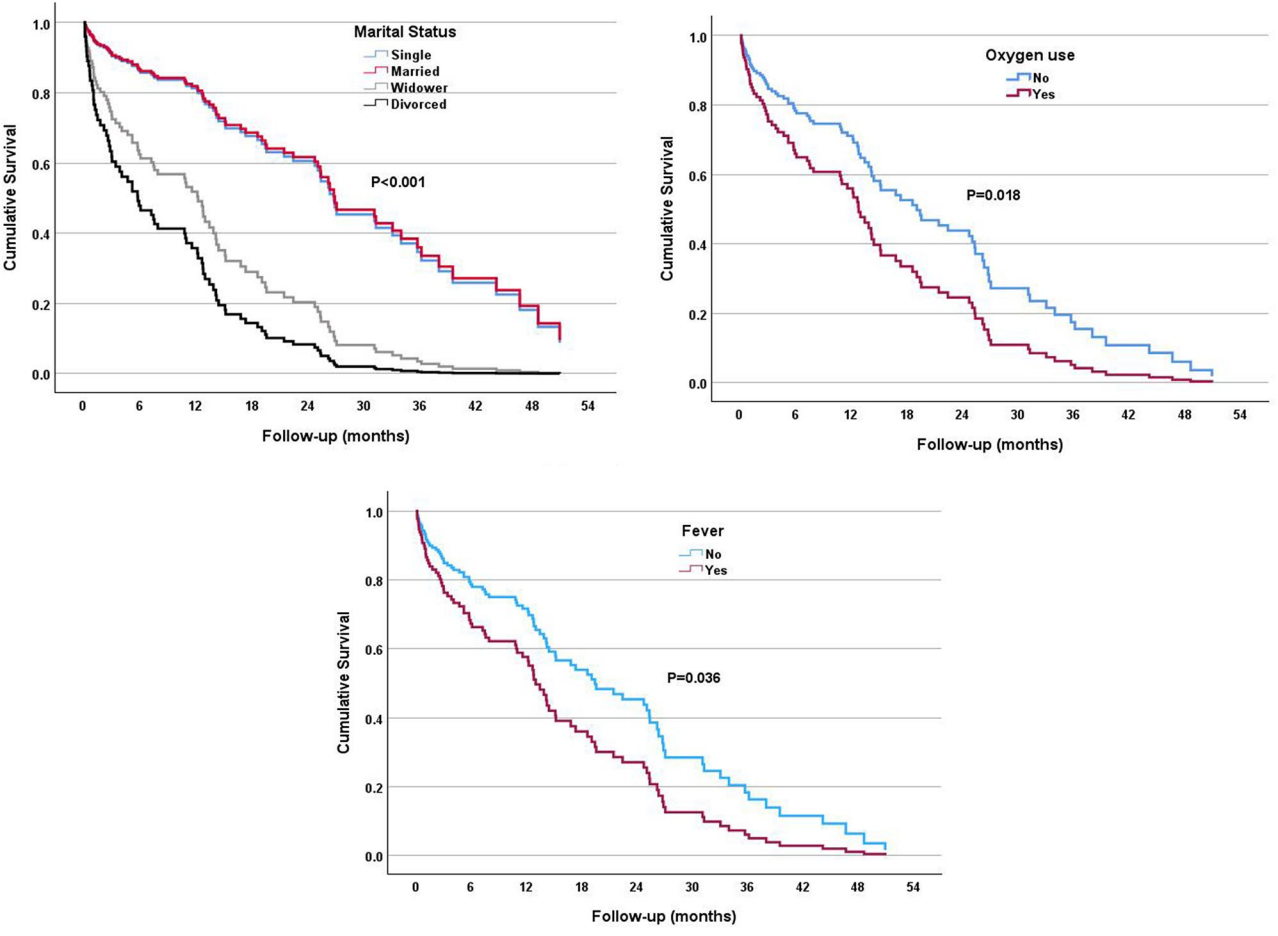
Cox proportional survival curves for mortality risk factors during follow-up: multivariable analysis results. * * Models adjusted for sex, age at feeding, marital status, pressure ulcer, hypothyroidism, oxygen use, fever, and cause of hospitalization: sepsis and neoplasm

Table 2, presents the selected blood test results of the patients and their association with mortality rate in a univariate analysis. The results demonstrate a significant association of the mortality rate with RDW (HR = 1.06, 95% CI [1.01–1.12], *p* = 0.022). In contrast, no statistically significant associations with mortality rate were found for other hematological and biochemical laboratory data including nutritional parameters such as hemoglobin, lymphocytes, albumin, creatinine, vitamins, and iron. During the follow-up period starting from the time of feeding tube insertion (that was before, during, or after the hospitalization) several parameters had a significant correlation to mortality (Fig. 3). Widowed individuals exhibited a striking higher mortality rate (HR = 3.11, 95% CI [1.83–5.26], *p* < 0.001), while divorced individuals faced an even more substantial higher risk compared to the reference category (HR = 4.63, 95% CI [1.98–10.81], *p* < 0.001). Individuals admitted to the hospital due to sepsis had a striking higher mortality rate (HR = 9.90, 95% CI [2.12–46.17], *p* = 0.004), and those with neoplasm faced a relatively high mortality rate (HR = 5.34, 95% CI [2.15–13.25], *p* < 0.001), however, the number of patients was very small. Furthermore, patients with fever and the use of oxygen exhibited a higher risk of mortality (HR = 1.65, 95% CI [1.03–2.65], *p* = 0.036, and HR = 1.82, 95% CI [1.11–2.98], *p* = 0.018) respectively.

**Table 2:** Association of admission blood test results with mortality: univariate survival analysis results

		Total				Alive				Death					95%CI		
		N	Median	25th	75th	N	Median	25th	75th	N	Median	25th	75th	**HR**	**lower**	**upper**	**P**
WBC	10^3^/µl	121	9.6	7.9	12.0	33	9.4	7.9	11.2	88	9.7	7.8	12.2	1.00	0.97	1.03	0.833
Hemoglobin	g/dl	121	10.6	9.5	12.1	33	11.1	10.0	12.4	88	10.5	9.5	12.0	1.01	0.90	1.13	0.871
Lymph TN	10^3^/µl	120	1.5	1.2	1.9	33	1.5	1.3	2.0	87	1.5	1.2	1.9	0.83	0.65	1.08	0.162
Neutr TN	10^3^/µl	120	7.3	4.9	9.4	33	6.9	3.8	9.6	87	7.6	5.1	9.4	1.01	0.97	1.04	0.752
Platlates	10^3^/µl	121	278.0	212.0	339.0	33	294.0	228.0	333.0	88	273.0	194.0	346.0	1.00	1.00	1.00	0.496
RDW	%	121	15.1	14.2	17.0	33	14.5	14.0	16.7	88	15.3	14.2	17.1	1.06	1.01	1.12	**0.022**
MCH	pg	121	29.1	27.3	30.3	33	29.2	27.9	30.3	88	29.1	26.5	30.4	1.00	1.00	1.01	0.418
MCV	fl.	121	90.1	86.1	93.3	33	91.5	88.8	93.1	88	89.5	84.7	93.4	0.99	0.96	1.02	0.432
Cholesterol	mg/dl	103	140.0	120.0	170.0	32	134.0	120.5	171.5	71	143.0	120.0	170.0	1.00	1.00	1.01	0.501
Albumin	g/dl	119	3.1	2.8	3.5	33	3.2	3.0	3.7	86	3.1	2.7	3.4	0.90	0.61	1.34	0.613
Total Protein	g/dl	119	6.3	5.8	6.9	33	6.7	5.9	7.2	86	6.2	5.7	6.9	1.00	1.00	1.00	0.563
Ferrum	mcg/dL	28	30.0	23.5	40.5	4	23.5	18.0	32.0	24	32.0	24.5	41.5	1.02	0.99	1.04	0.190
Vit.B-12	pg/mL	96	840.0	539.0	1240.5	28	894.8	530.0	1368.5	68	802.0	541.5	1234.0	1.00	1.00	1.00	0.166
TSH	mU/L	107	2.2	1.5	3.5	33	2.4	1.4	4.7	74	2.1	1.5	3.1	1.02	0.99	1.05	0.210
AST	U/l	111	20.0	15.0	27.0	30	20.0	16.0	25.0	81	19.6	15.0	27.0	1.01	1.00	1.01	0.159
ALT	U/l	117	17.0	11.0	25.0	32	17.5	12.0	24.5	85	17.0	11.0	25.0	1.00	0.99	1.01	0.553
ALP	U/l	118	96.5	77.0	124.0	33	100.0	85.0	119.0	85	94.0	67.0	125.0	1.00	1.00	1.00	0.808
Triglycerides	mg/dl	105	115.0	91.0	154.0	32	119.5	92.0	165.0	73	115.0	82.0	144.0	1.00	1.00	1.00	0.498
Creatinine	mg/dl	122	0.7	0.5	1.0	34	0.8	0.6	1.0	88	0.7	0.5	1.0	1.16	0.68	1.97	0.586
Urea	mg/dl	122	54.0	38.0	76.0	34	52.0	38.0	80.0	88	54.0	37.5	75.0	1.00	0.99	1.01	0.858
Phosphorus	mg/dl	102	3.2	2.7	3.7	33	3.7	2.9	3.8	69	3.0	2.6	3.6	0.72	0.50	1.03	0.069

*Abbreviations* ALP, alkaline phosphatase; ALT, alanine transaminase; AST, aspartate aminotransferase; MCH, mean cell hemoglobin; MCV, mean cell volume; RDW, red cell distribution width; TN, total number; WBC, *white blood count*

Additionally, higher RDW levels correlated with a high mortality rate (HR = 1.08, 95% CI [1.02–1.15], *p* = 0.011). On the contrary, individuals with hypothyroidism showed a lower risk of mortality (HR = 0.45, 95% CI [0.25–0.80], *p* = 0.007).

Table 3 presents the one-year follow-up multivariant survival analysis specifically focusing on outcomes following feeding tube insertion. In this analysis, similar trends were observed. Men, widowed, and divorced individuals exhibited significantly elevated risks of mortality (HR = 3.18, 95% CI [1.64–6.16], *p* = 0.001), HR = 3.37, 95% CI [1.67–6.81], *p* = 0.001), and (HR = 5.26, 95% CI [1.80–15.38], *p* = 0.002) respectively. Neoplasm was once again associated with a notably higher risk of mortality (HR = 5.60, 95% CI [2.00–15.69], *p* = 0.001), however, the number of patients was low. Notably, RDW levels exhibited an increased risk of mortality (HR = 1.08, 95% CI [1.00–1.17], *p* = 0.048), while hypothyroidism and low Lymphocyte percentage showed a decreased risk (HR = 0.37, 95% CI [0.15–0.95], *p* = 0.040) and (HR = 0.93, 95% CI [0.88–0.98], *p* = 0.005) respectively. Pressure ulcers and MCHC did not demonstrate statistically significant associations with mortality risk in the multivariant analysis.

**Table 3:** Risk factors for mortality in the first year of follow-up: multivariable analysis results

12 months follow-up	Exp(B)	95.0% CI for Exp(B)		Sig.
		Lower	Upper	
Men	3.18	1.64	6.16	0.001
Widower	3.37	1.67	6.81	0.001
Divorced	5.26	1.80	15.38	0.002
Neoplasm	5.60	2.00	15.69	0.001
Pressure Ulcers	2.08	0.92	4.71	0.079
Hypothyroidism	0.37	0.15	0.95	0.040
RDW	1.08	1.00	1.17	0.048
Lymph%	0.93	0.88	0.98	0.005

*Abbreviations* RDW, red cell distribution width

## Discussion

In a prior study, we demonstrated a high in-hospital mortality rate (60.5%) among older adults who initiated enteral feeding during their hospitalization in internal medicine wards of a general hospital [10]. Geriatric assessment was proposed to have a protective effect while pressure sores, lymphopenia, and lower serum cholesterol levels were mortality risk factors.

The present study provides additional insights into factors and measurements influencing mortality rates among older patients with enteral feeding in a Geriatric Medical Center. A total cumulative duration of 1911 patients months allows us to assess the long-term impact and feasibility of feeding tube interventions, aiding clinical decision-making and patient counseling. The in-hospital mortality rate of older adults admitted to a geriatric hospital receiving or starting EN was significantly lower (33%( than in the general hospital settings. This is probably due to the benefit of a comprehensive geriatric assessment of all patients and the less acute nature of the patients in the geriatric center.

A clear link between marital status and mortality rates was highlighted. Divorced and widowed individuals faced higher mortality risks compared to their married counterparts. This underscores the vital role of strong support systems in overall well-being, potentially leading to better health outcomes through increased emotional and practical assistance. Prior studies have demonstrated that married older adults tend to live longer [17–19]. These findings appear to apply to patients receiving enteral feeding as well, likely for similar reasons. Trends toward higher mortality rates were observed in patients with fever or infectious disease as the main cause of hospitalization but the number of patients was too low to reach statistical significance. This may also imply a grim prognosis of tube feeding in acute settings. As in the prior study [10], a univariate analysis linked pressure ulcers to increased hospital mortality. Pressure ulcers were not associated with mortality in the multivariate analysis perhaps indicating they are merely indicators of a worse baseline condition nutritional and functional status and frailer patients [20, 21]. Oxygen use had similar results of a worse prognosis only in univariate analysis, perhaps due to a higher aspiration rate in these patients resulting in frequent oxygen use or again because it implies a worse baseline. Laboratory biomarkers of malnutrition such as albumin, cholesterol, hemoglobin, platelets, and lymphocytes [22–25] did not show significant differences, however, an association between RDW and mortality rates was established. In a recent study [26], Haenggi et al. found that among medical patients at nutritional risk, RDW correlated with several nutritional parameters and served as a strong prognostic marker for both short- and long-term adverse clinical outcomes. Our findings strengthen this result, emphasizing the need for further research regarding the use of RDW as a marker of malnutrition and inflammation in frail older adults. Interestingly, the significant factors correlating with mortality rate during the first year (marital status, pressure ulcers, and RDW) persisted throughout the entire study period.

There is limited research supporting the consistent benefits of enteral feeding, such as improved biochemical markers, weight gain, and various clinical measures, especially in older adults with comorbidities and advanced illnesses. Additionally, literature about enteral feeding’s impact on mortality in older individuals is limited. In prior studies, tube feeding didn’t enhance survival, particularly in advanced dementia cases, and early tube insertion did not yield better outcomes or improve the quality of life [7, 8, 27]. In many cases, inserting a feeding tube requires medical treatment and supervision to ensure patient comfort and prevent them from attempting to remove the tube due to discomfort or confusion [28]. Despite this evidence, tube feeding remains a reliable and widely utilized approach, particularly for older patients with acute conditions such as impaired consciousness, dysphagia, respiratory failure, or severe malnutrition. Its effectiveness in providing short-term EN offers noticeable advantages for specific patients, making it a valuable tool in acute care settings [29, 30]. Enteral feeding requires proper consent and consideration of the patient’s best interests however, many patients in need of enteral feeding cannot give informed consent. Advanced care planning may provide aid for decision making enabling better personalized individual care. Unfortunately, while Israeli law mandates providing nutritional support in the absence of care planning, only 0.3% of older adults in Israel had Advance Care Planning documentation true to 2013 [31] as opposed to 33% of the US public [32, 33]. This with other ethical, and religious factors, alongside the collective memory linked to the Holocaust, causes Feeding tube usage in Israel to be notably higher than in other countries [34, 35].

Most studies addressing the prognosis of older patients on enteral tube feeding were performed in ambulatory and geriatric care settings [36–40] and focused on long-term mortality in specific disorders such as dementia [38, 39], stroke [41, 42], and cancer [42, 43]. the results of this study found a relatively long duration of tube feeding with a median follow-up duration of more than one year. This is an indication that in selected patients enteral feeding may prolong life. However, the prognosis throughout the study period was glim with a 73% mortality rate. Furthermore, a low Norton scale, high comorbidity burden, and poly-pharmacy suggest a low quality of life [44] for most participants.

Nutritional status assessed by the SNAQ score did not affect mortality for either new or chronic EN. Information regarding the effect of EN on mortality and nutritional status in older adults with a high comorbid burden is limited [9], with recommendations mostly based on expert opinions [45]. Since all patients in this study received EN and most had a low SNAQ score, assessing the impact of EN on nutritional status was beyond the scope of this study. The high mortality rate among older hospitalized adults, most of whom had low SNAQ scores, raises the question of how much emphasis should be placed on nutritional status in this population. Our hospital does not have surgical or ICU wards and thus indications for EN are due to chronic conditions such as malnutrition, dehydration, aspirations, or swallowing problems. Although 42 patients began EN during their hospitalization, no differences in mortality were found between them and patients with pre-hospitalization EN. This suggests that the need for EN in these older patients indicates a poor prognosis, regardless of the duration of EN before hospitalization. Besides its retrospective nature, this study has several limitations. It was performed in a single center and variability regarding the tube insertion and feeding may exist. Precise functional and cognitive status was not assessed, however, as described we believe the data provided is enough to understand the nature of most participants. The medical center accepts patients from different departments. This may affect mainly short-term results. Most mortality risk factors did not change in the long-term follow-up indicating the nature of hospitalization did not have a major effect on the outcomes.

## Conclusion

Older adults in a geriatric medical center receiving EN have better short-term outcomes than those in a general hospital. However, despite comprehensive multidisciplinary geriatric care, the long-term outcomes of tube feeding remain poor, with high mortality and probably low quality of life. Marital status was significantly linked to mortality, implying the importance of strong support systems in the geriatric population. Additionally, oxygen use and RDW may have prognostic value for both short- and long-term outcomes. Patients and caregivers should be made aware of these factors when making decisions about tube feeding and during discussions on advanced care planning.

## Data Availability

The datasets generated and analyzed during the current study are not publicly available due to privacy and ethical restrictions but are available from the corresponding author on reasonable request.
